# Effects of cigarette smoking on priapism induced by quetiapine: a case report

**DOI:** 10.1186/2008-2231-20-55

**Published:** 2012-10-15

**Authors:** Seyed Hamzeh Hosseini, Hafez Bajoghli, Padideh Ghaeli

**Affiliations:** 1 Behavioral Sciences and Psychiatry Research Center, Zare Psychiatry Hospital, Mazandaran Medical Sciences University, Sari, Iran; 2 Psychiatry & Psychology Research Center (PPRC), Roozbeh Hospital, Tehran University of medical Sciences, Tehran, Iran; 3 Research Center for Rational Use of drugs, Faculty of Pharmacy, and Roozbeh Hospital, Tehran University of medical Sciences (TUMS), South Kargar St, 1333795914, Tehran, Iran

**Keywords:** Priapism, Antipsychotic, Quetiapine, Cigarette smoking

## Abstract

Priapism is defined as an unwanted, prolonged, and painful erection which is unrelated to sexual stimulation. Some case studies suggest that priapism is an adverse effect of antipsychotic medications. In our case study a 30 year-old Iranian male with schizophrenia was experiencing recurrent priapism associated with quetiapine use. There are three interesting facts about this case: Firstly, the patient suffered priapism after even low dose consumption of quetiapine. Secondly, this case had experienced priapism with risperidone, olanzapine, and even clozapine in the past, suggesting a possible pharmacodynamic interaction of antipsychotics and inner biological traits in this particular case. Thirdly, priapism induced by low dose quetiapine was resolved after cigarette smoking.

## Introduction

The etiology of priapism may be idiopathic, drug-induced, or related to some medical problems like sickle cell disease or leukemia [1]. Priapism has been notenoted as an adverse effect of both first- and second-generation anti psychotic medications [2]. Peripheral alpha-1 adrenergic blockade has been considered as the main cause of priapism [3]. Trazodone with its alpha-1 antagonistic properties, has been reported to induce priapism [4]. Antipsychotics are accounted for 15-26% of priapism cases that are induced by medications [5]. Previous case reports of priapism associated with quetiapine were those with a total daily dose of 600mg or greater [4,6-9]. One case involved a single overdose of 675mg [8]. Presently, we report a case of priapism associated with 25mg use of quetiapine [10].

## Case Presentation

A 30 year old single unemployed man was presented to our out-patient psychiatry clinic because of a recurrence of the psychotic episode. The patient did not have any comorbidity with medical diseases or substance abuse disorders in the past. Recurrence of the psychotic episode was due to the self-discontinuation of quetiapine secondary to recurrent priapism. He was diagnosed with paranoid schizophrenia since 10 years ago. His main symptoms included delusions of control, persecutory delusion, and auditory hallucination. His symptoms were relatively controlled. He had several recurrences and all of them were associated with drug discontinuation due to the patient's lack of compliance. He did not have any major stressors before recurrences. He was under treatment of first- and second-generation antipsychotics. He developed drug-induced Parkinsonism several times which was resolved with dose-adjustment and anti cholinergic therapy. The patient experienced priapism secondary to use of some antipsychotic medications like risperidone, olanzapine, clozapine and finally quetiapine. The diagnosis of priapism was confirmed by a urologic consult. All medical causes of priapism were ruled-out by a thorough urological history and physical examination. To prevent priapism, we substituted the combination therapy of lithium and sodium-valproate for control of psychotic symptoms. After six months he again experienced psychotic symptoms. Then, we started a low-dose quetiapine (25mg per day, gradually increased to 100mg per day). After 2months of this regimen, he experienced a 6- hour-long painful priapism. It was resolved with diazepam use without any surgical intervention. Despite reducing the quetiapine dose to 25mg per day, he experienced priapism every 4–5days. Astonishingly, he did not experience priapism with accidental cigarette smoking. As mentioned above, the patient did not have a history of substance abuse disorder. He accidentally and adaptively smoked cigarette to get rid of priapism. After a two-week challenge test of smoking cessation, he re-experienced priapism again. After all the challenges and even though smoking is considered a maladaptive behavior to get rid of the side effects, the patient decided to keep on smoking while consuming quetiapine. He has not been complaining of priapism since then. Patient signed a consent form for publication of this report.

## Discussion

In a few cases quetiapine has been described as the cause of priapism [1-5], which may be due to its intermediate affinity for alpha-1 adrenoceptors as compared with other atypical antipsychotic medications [11]. Notably, some cases have even suggested using quetiapine for patients who are at risk for developing priapism [9]. An interesting fact about this case is the relationship between smoking cessation and recurrence of priapism. It is well-known that cigarette smoking induces cytochrome P450 1A2 (CYP1A2) and as a result it may decreases the serum level of some drugs like olanzapine and clozapine that are CYP1A2 substrates [12]. However, since quetiapine is not a known CYP1A2 substrate, the effect of cigarette smoking on this drug may be due to an unknown interaction. One of the limitations of our study was that we did not check the serum level of quetiapine before and after smoking. Therefore, we do not have any valid evidence that shows the changes of quetiapine concentration with cigarette smoking. Another limitation of this study is the lack of an objectified scoring system to show the effect size of this correlation. Thus, the presented report leads to a correlation between cigarette smoking and disappearing of priapism in this case rather than causality between the variables. Certain aspects of this case merit further comment and comparison to other reported cases. In the present case, the patient received daily doses of quetiapine far lower than 600mg that was given in other studies [5,8,13-16]. Furthermore, this patient had experienced priapism with different types of antipsychotics. Therefore, the authors suggest that this patient may have a biological and/or genetic tendency to develop drug-induced priapism. Further studies on the connection between biological tendencies and drug-induced priapism are recommended.

## Competing interests

All three authors declare that they have no competing interests.

## Authors’ contributions

Dr Hosseini was the psychiatrist in charge of the patient; he prepared and wrote the original data. Dr Bajoghli was involved in following the patient and editing the manuscript. Dr Ghaeli was given the pharmacotherapy consult for managing the patient properly and was involved in editing, revising and submitting the manuscript. All authors read and approved the final manuscript.
